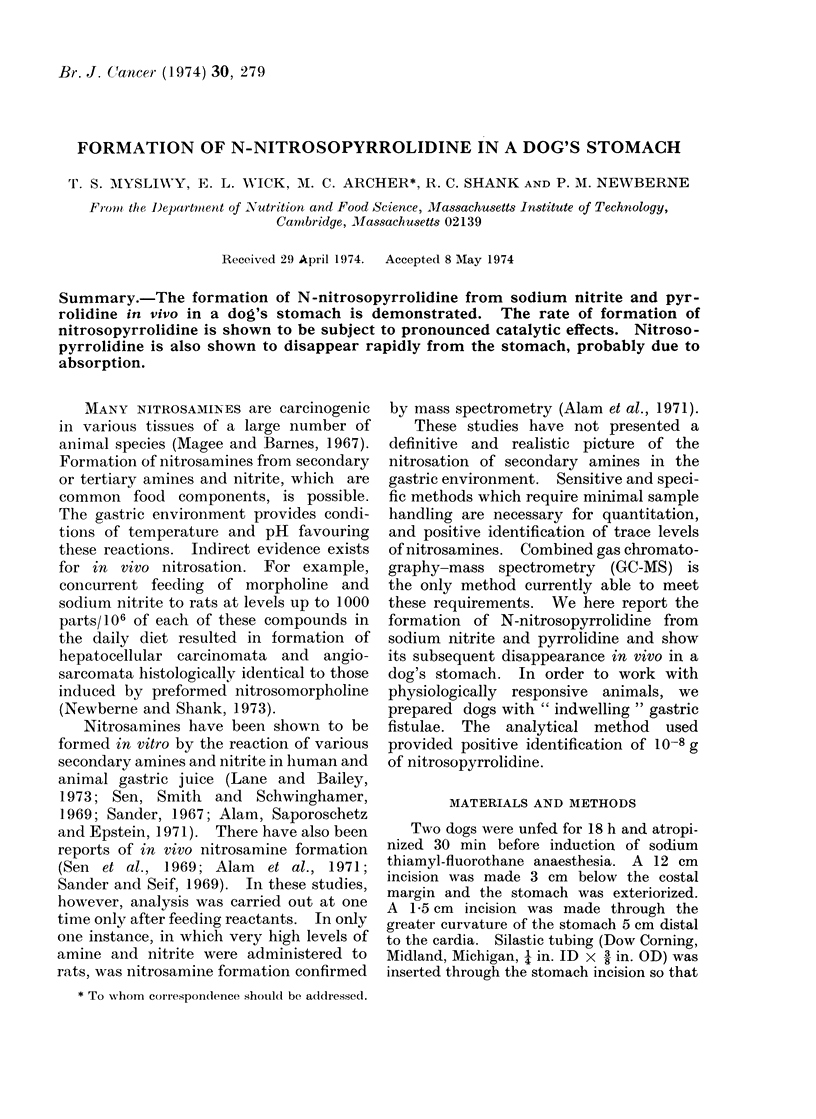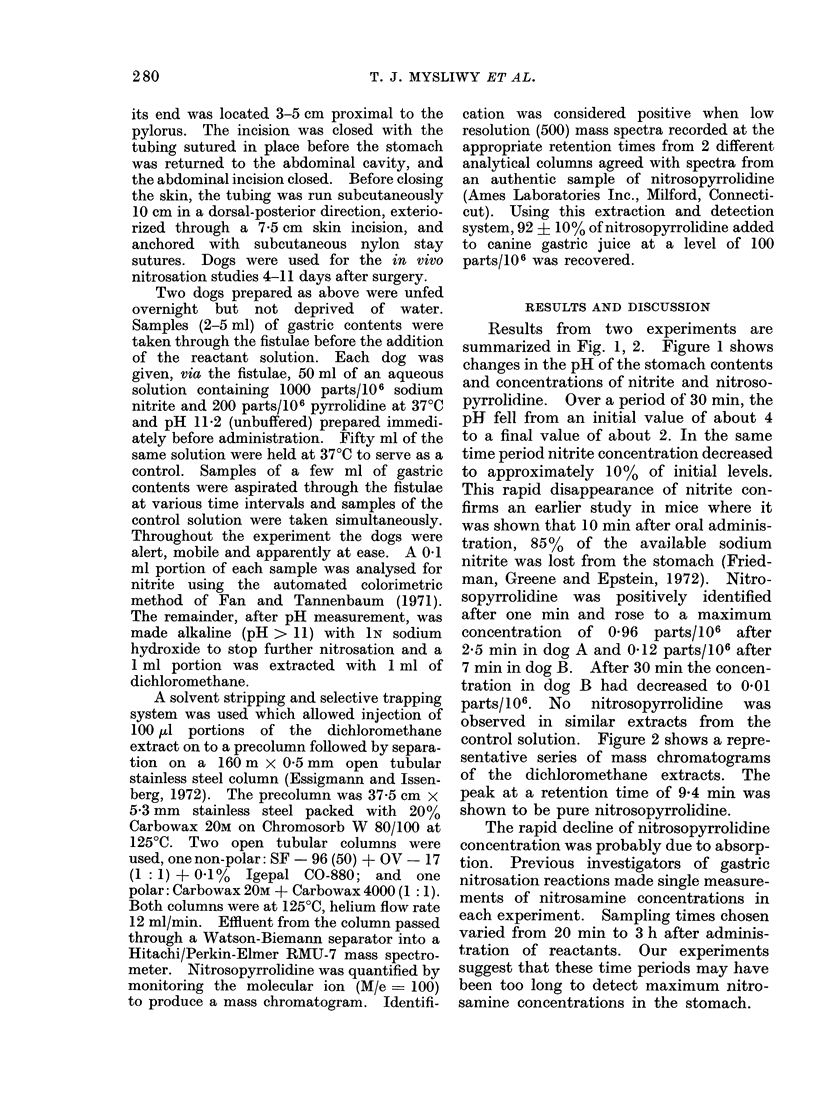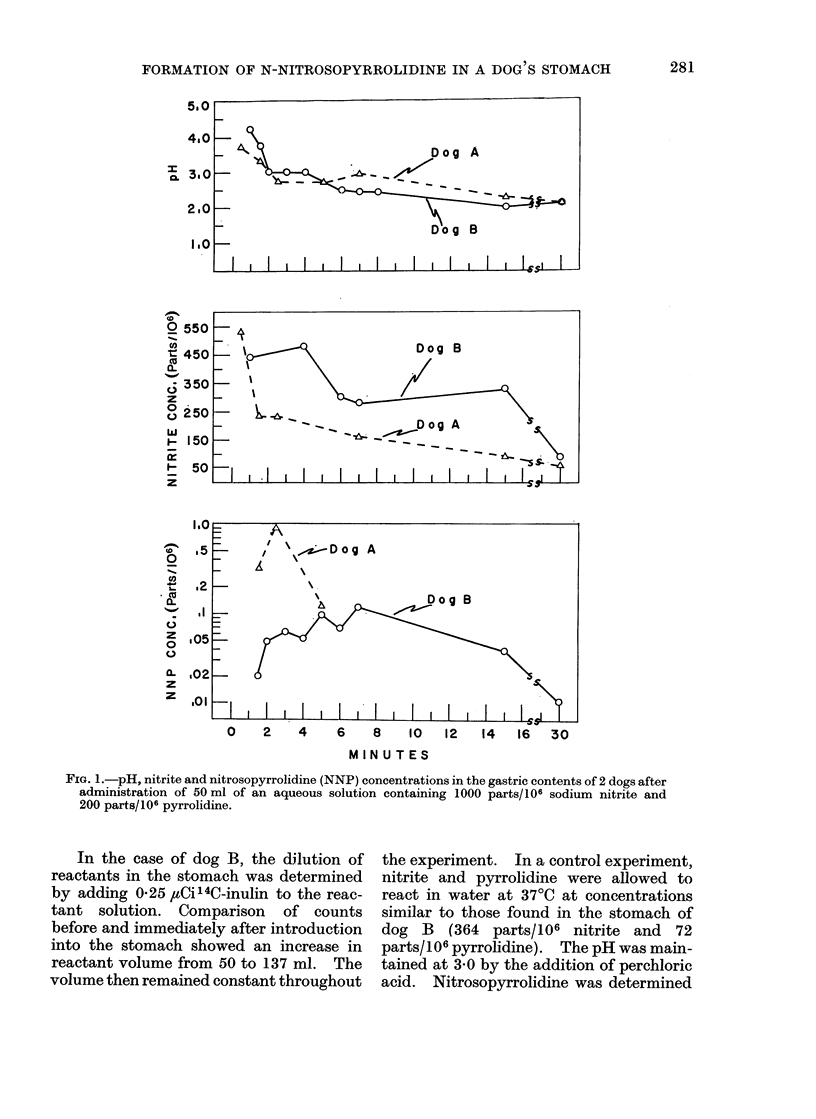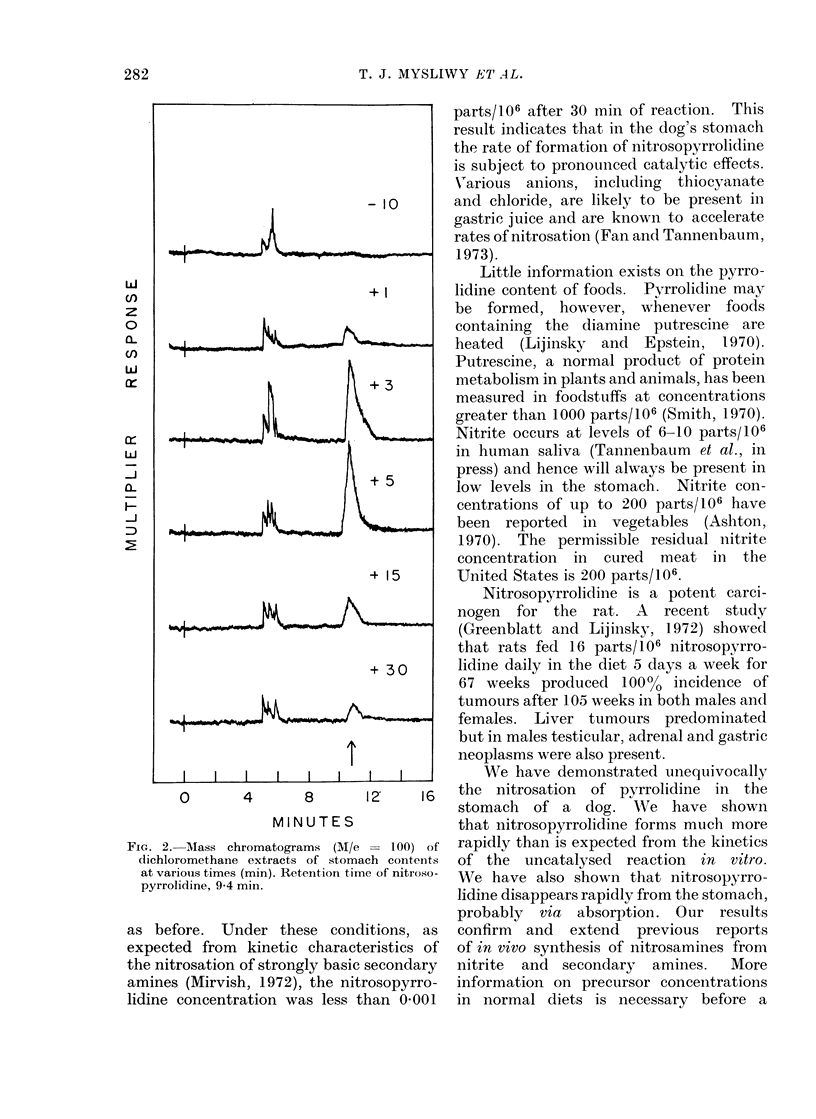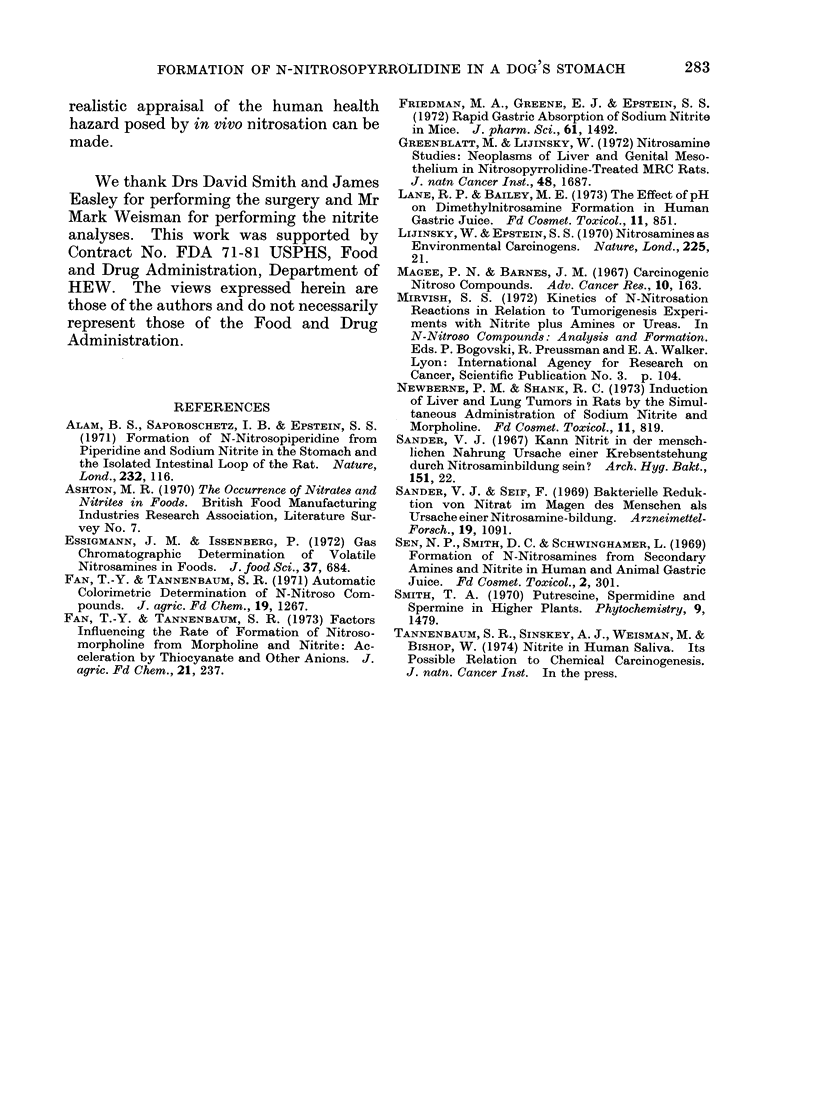# Formation of N-Nitrosopyrrolidine in a Dog's Stomach

**DOI:** 10.1038/bjc.1974.192

**Published:** 1974-09

**Authors:** T. S. Mysliwy, E. L. Wick, M. C. Archer, R. C. Shank, P. M. Newberne

## Abstract

The formation of N-nitrosopyrrolidine from sodium nitrite and pyrrolidine *in vivo* in a dog's stomach is demonstrated. The rate of formation of nitrosopyrrolidine is shown to be subject to pronounced catalytic effects. Nitrosopyrrolidine is also shown to disappear rapidly from the stomach, probably due to absorption.


*Br. J. Cancer* (1974) **30**, 279

### FORMATION OF N-NITROSOPYRROLIDINE IN A DOG'S STOMACH

T. S. MYSLIWY, E. L. WICK, M. C. ARCHER*, R. C. SHANK AND P. M. NEWBERNE

*From the Department of Nutrition and Food Science, Massachusetts Institute of Technology, Cambridge, Massachusetts* 02139

Received 29 April 1974. Accepted 8 May 1974

**Summary.—The formation of N-nitrosopyrrolidine from sodium nitrite and pyrrolidine *in vivo* in a dog's stomach is demonstrated. The rate of formation of nitrosopyrrolidine is shown to be subject to pronounced catalytic effects. Nitrosopyrrolidine is also shown to disappear rapidly from the stomach, probably due to absorption.**

MANY NITROSAMINES are carcinogenic in various tissues of a large number of animal species (Magee and Barnes, 1967). Formation of nitrosamines from secondary or tertiary amines and nitrite, which are common food components, is possible. The gastric environment provides conditions of temperature and pH favouring these reactions. Indirect evidence exists for *in vivo* nitrosation. For example, concurrent feeding of morpholine and sodium nitrite to rats at levels up to 1000 parts/$10^6$ of each of these compounds in the daily diet resulted in formation of hepatocellular carcinomata and angiosarcomata histologically identical to those induced by preformed nitrosomorpholine (Newberne and Shank, 1973).

Nitrosamines have been shown to be formed *in vitro* by the reaction of various secondary amines and nitrite in human and animal gastric juice (Lane and Bailey, 1973; Sen, Smith and Schwinghamer, 1969; Sander, 1967; Alam, Saporoschetz and Epstein, 1971). There have also been reports of *in vivo* nitrosamine formation (Sen *et al.*, 1969; Alam *et al.*, 1971; Sander and Seif, 1969). In these studies, however, analysis was carried out at one time only after feeding reactants. In only one instance, in which very high levels of amine and nitrite were administered to rats, was nitrosamine formation confirmed by mass spectrometry (Alam *et al.*, 1971).

These studies have not presented a definitive and realistic picture of the nitrosation of secondary amines in the gastric environment. Sensitive and specific methods which require minimal sample handling are necessary for quantitation, and positive identification of trace levels of nitrosamines. Combined gas chromatography–mass spectrometry (GC-MS) is the only method currently able to meet these requirements. We here report the formation of N-nitrosopyrrolidine from sodium nitrite and pyrrolidine and show its subsequent disappearance *in vivo* in a dog's stomach. In order to work with physiologically responsive animals, we prepared dogs with " indwelling " gastric fistulae. The analytical method used provided positive identification of $10^{-8}$ g of nitrosopyrrolidine.

### MATERIALS AND METHODS

Two dogs were unfed for 18 h and atropinized 30 min before induction of sodium thiamyl-fluorothane anaesthesia. A 12 cm incision was made 3 cm below the costal margin and the stomach was exteriorized. A 1·5 cm incision was made through the greater curvature of the stomach 5 cm distal to the cardia. Silastic tubing (Dow Corning, Midland, Michigan, $\frac{1}{4}$ in. ID × $\frac{3}{8}$ in. OD) was inserted through the stomach incision so that

* To whom correspondence should be addressed.

its end was located 3–5 cm proximal to the pylorus. The incision was closed with the tubing sutured in place before the stomach was returned to the abdominal cavity, and the abdominal incision closed. Before closing the skin, the tubing was run subcutaneously 10 cm in a dorsal-posterior direction, exteriorized through a 7·5 cm skin incision, and anchored with subcutaneous nylon stay sutures. Dogs were used for the *in vivo* nitrosation studies 4–11 days after surgery.

Two dogs prepared as above were unfed overnight but not deprived of water. Samples (2–5 ml) of gastric contents were taken through the fistulae before the addition of the reactant solution. Each dog was given, *via* the fistulae, 50 ml of an aqueous solution containing 1000 parts/$10^6$ sodium nitrite and 200 parts/$10^6$ pyrrolidine at 37°C and pH 11·2 (unbuffered) prepared immediately before administration. Fifty ml of the same solution were held at 37°C to serve as a control. Samples of a few ml of gastric contents were aspirated through the fistulae at various time intervals and samples of the control solution were taken simultaneously. Throughout the experiment the dogs were alert, mobile and apparently at ease. A 0·1 ml portion of each sample was analysed for nitrite using the automated colorimetric method of Fan and Tannenbaum (1971). The remainder, after pH measurement, was made alkaline (pH $>$ 11) with 1N sodium hydroxide to stop further nitrosation and a 1 ml portion was extracted with 1 ml of dichloromethane.

A solvent stripping and selective trapping system was used which allowed injection of 100 $\mu$l portions of the dichloromethane extract on to a precolumn followed by separation on a 160 m $\times$ 0·5 mm open tubular stainless steel column (Essigmann and Issenberg, 1972). The precolumn was 37·5 cm $\times$ 5·3 mm stainless steel packed with 20% Carbowax 20M on Chromosorb W 80/100 at 125°C. Two open tubular columns were used, one non-polar: SF — 96 (50) + OV — 17 (1 : 1) + 0·1% Igepal CO-880; and one polar: Carbowax 20M + Carbowax 4000 (1 : 1). Both columns were at 125°C, helium flow rate 12 ml/min. Effluent from the column passed through a Watson-Biemann separator into a Hitachi/Perkin-Elmer RMU-7 mass spectrometer. Nitrosopyrrolidine was quantified by monitoring the molecular ion (M/e = 100) to produce a mass chromatogram. Identification was considered positive when low resolution (500) mass spectra recorded at the appropriate retention times from 2 different analytical columns agreed with spectra from an authentic sample of nitrosopyrrolidine (Ames Laboratories Inc., Milford, Connecticut). Using this extraction and detection system, 92 $\pm$ 10% of nitrosopyrrolidine added to canine gastric juice at a level of 100 parts/$10^6$ was recovered.

### RESULTS AND DISCUSSION

Results from two experiments are summarized in Fig. 1, 2. Figure 1 shows changes in the pH of the stomach contents and concentrations of nitrite and nitrosopyrrolidine. Over a period of 30 min, the pH fell from an initial value of about 4 to a final value of about 2. In the same time period nitrite concentration decreased to approximately 10% of initial levels. This rapid disappearance of nitrite confirms an earlier study in mice where it was shown that 10 min after oral administration, 85% of the available sodium nitrite was lost from the stomach (Friedman, Greene and Epstein, 1972). Nitrosopyrrolidine was positively identified after one min and rose to a maximum concentration of 0·96 parts/$10^6$ after 2·5 min in dog A and 0·12 parts/$10^6$ after 7 min in dog B. After 30 min the concentration in dog B had decreased to 0·01 parts/$10^6$. No nitrosopyrrolidine was observed in similar extracts from the control solution. Figure 2 shows a representative series of mass chromatograms of the dichloromethane extracts. The peak at a retention time of 9·4 min was shown to be pure nitrosopyrrolidine.

The rapid decline of nitrosopyrrolidine concentration was probably due to absorption. Previous investigators of gastric nitrosation reactions made single measurements of nitrosamine concentrations in each experiment. Sampling times chosen varied from 20 min to 3 h after administration of reactants. Our experiments suggest that these time periods may have been too long to detect maximum nitrosamine concentrations in the stomach.

FIG. 1.—pH, nitrite and nitrosopyrrolidine (NNP) concentrations in the gastric contents of 2 dogs after administration of 50 ml of an aqueous solution containing 1000 parts/$10^6$ sodium nitrite and 200 parts/$10^6$ pyrrolidine.

In the case of dog B, the dilution of reactants in the stomach was determined by adding 0·25 $\mu$Ci$^{14}$C-inulin to the reactant solution. Comparison of counts before and immediately after introduction into the stomach showed an increase in reactant volume from 50 to 137 ml. The volume then remained constant throughout the experiment. In a control experiment, nitrite and pyrrolidine were allowed to react in water at 37°C at concentrations similar to those found in the stomach of dog B (364 parts/$10^6$ nitrite and 72 parts/$10^6$ pyrrolidine). The pH was maintained at 3·0 by the addition of perchloric acid. Nitrosopyrrolidine was determined

FIG. 2.—Mass chromatograms (M/e = 100) of dichloromethane extracts of stomach contents at various times (min). Retention time of nitrosopyrrolidine, 9·4 min.

as before. Under these conditions, as expected from kinetic characteristics of the nitrosation of strongly basic secondary amines (Mirvish, 1972), the nitrosopyrrolidine concentration was less than 0·001 parts/$10^6$ after 30 min of reaction. This result indicates that in the dog's stomach the rate of formation of nitrosopyrrolidine is subject to pronounced catalytic effects. Various anions, including thiocyanate and chloride, are likely to be present in gastric juice and are known to accelerate rates of nitrosation (Fan and Tannenbaum, 1973).

Little information exists on the pyrrolidine content of foods. Pyrrolidine may be formed, however, whenever foods containing the diamine putrescine are heated (Lijinsky and Epstein, 1970). Putrescine, a normal product of protein metabolism in plants and animals, has been measured in foodstuffs at concentrations greater than 1000 parts/$10^6$ (Smith, 1970). Nitrite occurs at levels of 6–10 parts/$10^6$ in human saliva (Tannenbaum *et al.*, in press) and hence will always be present in low levels in the stomach. Nitrite concentrations of up to 200 parts/$10^6$ have been reported in vegetables (Ashton, 1970). The permissible residual nitrite concentration in cured meat in the United States is 200 parts/$10^6$.

Nitrosopyrrolidine is a potent carcinogen for the rat. A recent study (Greenblatt and Lijinsky, 1972) showed that rats fed 16 parts/$10^6$ nitrosopyrrolidine daily in the diet 5 days a week for 67 weeks produced 100% incidence of tumours after 105 weeks in both males and females. Liver tumours predominated but in males testicular, adrenal and gastric neoplasms were also present.

We have demonstrated unequivocally the nitrosation of pyrrolidine in the stomach of a dog. We have shown that nitrosopyrrolidine forms much more rapidly than is expected from the kinetics of the uncatalysed reaction *in vitro*. We have also shown that nitrosopyrrolidine disappears rapidly from the stomach, probably *via* absorption. Our results confirm and extend previous reports of *in vivo* synthesis of nitrosamines from nitrite and secondary amines. More information on precursor concentrations in normal diets is necessary before a realistic appraisal of the human health hazard posed by *in vivo* nitrosation can be made.

We thank Drs David Smith and James Easley for performing the surgery and Mr Mark Weisman for performing the nitrite analyses. This work was supported by Contract No. FDA 71-81 USPHS, Food and Drug Administration, Department of HEW. The views expressed herein are those of the authors and do not necessarily represent those of the Food and Drug Administration.

### REFERENCES

ALAM, B. S., SAPOROSCHETZ, I. B. & EPSTEIN, S. S. (1971) Formation of N-Nitrosopiperidine from Piperidine and Sodium Nitrite in the Stomach and the Isolated Intestinal Loop of the Rat. *Nature, Lond.*, **232**, 116.

ASHTON, M. R. (1970) *The Occurrence of Nitrates and Nitrites in Foods.* British Food Manufacturing Industries Research Association, Literature Survey No. 7.

ESSIGMANN, J. M. & ISSENBERG, P. (1972) Gas Chromatographic Determination of Volatile Nitrosamines in Foods. *J. food Sci.*, **37**, 684.

FAN, T.-Y. & TANNENBAUM, S. R. (1971) Automatic Colorimetric Determination of N-Nitroso Compounds. *J. agric. Fd Chem.*, **19**, 1267.

FAN, T.-Y. & TANNENBAUM, S. R. (1973) Factors Influencing the Rate of Formation of Nitrosomorpholine from Morpholine and Nitrite: Acceleration by Thiocyanate and Other Anions. *J. agric. Fd Chem.*, **21**, 237.

FRIEDMAN, M. A., GREENE, E. J. & EPSTEIN, S. S. (1972) Rapid Gastric Absorption of Sodium Nitrite in Mice. *J. pharm. Sci.*, **61**, 1492.

GREENBLATT, M. & LIJINSKY, W. (1972) Nitrosamine Studies: Neoplasms of Liver and Genital Mesothelium in Nitrosopyrrolidine-Treated MRC Rats. *J. natn Cancer Inst.*, **48**, 1687.

LANE, R. P. & BAILEY, M. E. (1973) The Effect of pH on Dimethylnitrosamine Formation in Human Gastric Juice. *Fd Cosmet. Toxicol.*, **11**, 851.

LIJINSKY, W. & EPSTEIN, S. S. (1970) Nitrosamines as Environmental Carcinogens. *Nature, Lond.*, **225**, 21.

MAGEE, P. N. & BARNES, J. M. (1967) Carcinogenic Nitroso Compounds. *Adv. Cancer Res.*, **10**, 163.

MIRVISH, S. S. (1972) Kinetics of N-Nitrosation Reactions in Relation to Tumorigenesis Experiments with Nitrite plus Amines or Ureas. In *N-Nitroso Compounds: Analysis and Formation.* Eds. P. Bogovski, R. Preussman and E. A. Walker. Lyon: International Agency for Research on Cancer, Scientific Publication No. 3. p. 104.

NEWBERNE, P. M. & SHANK, R. C. (1973) Induction of Liver and Lung Tumors in Rats by the Simultaneous Administration of Sodium Nitrite and Morpholine. *Fd Cosmet. Toxicol.*, **11**, 819.

SANDER, V. J. (1967) Kann Nitrit in der menschlichen Nahrung Ursache einer Krebsentstehung durch Nitrosaminbildung sein? *Arch. Hyg. Bakt.*, **151**, 22.

SANDER, V. J. & SEIF, F. (1969) Bakterielle Reduktion von Nitrat im Magen des Menschen als Ursache einer Nitrosamine-bildung. *Arzneimettel-Forsch.*, **19**, 1091.

SEN, N. P., SMITH, D. C. & SCHWINGHAMER, L. (1969) Formation of N-Nitrosamines from Secondary Amines and Nitrite in Human and Animal Gastric Juice. *Fd Cosmet. Toxicol.*, **2**, 301.

SMITH, T. A. (1970) Putrescine, Spermidine and Spermine in Higher Plants. *Phytochemistry*, **9**, 1479.

TANNENBAUM, S. R., SINSKEY, A. J., WEISMAN, M. & BISHOP, W. (1974) Nitrite in Human Saliva. Its Possible Relation to Chemical Carcinogenesis. *J. natn. Cancer Inst.* In the press.